# Early Clinical and Radiological Experience with a Ceramic Bone Graft Substitute in the Treatment of Benign and Borderline Bone Lesions

**DOI:** 10.1038/s41598-018-33736-w

**Published:** 2018-10-18

**Authors:** Peter Frederik Horstmann, Werner Herbert Hettwer, Nicolai Stefan Kaltoft, Michael Mørk Petersen

**Affiliations:** 1 Musculoskeletal Tumor Section, Department of Orthopedic Surgery, Rigshospitalet, University of Copenhagen, Blegdamsvej 9, 2100 Copenhagen, Denmark; 2 Department of Radiology, Rigshospitalet, University of Copenhagen, Blegdamsvej 9, 2100 Copenhagen, Denmark

## Abstract

Substitutes for bone grafts experience increasing popularity, but the need for defect-filling following simple curettage of benign bone lesions is controversial. In this study, we wish to objectively report the radiological changes following bone defect-filling using a composite ceramic bone graft substitute, as well as the clinical results and complications. We evaluated 35 surgically treated benign bone lesions with subsequent defect-filling using two variants of a composite ceramic bone graft substitute (CERAMENT|BONE VOID FILLER or CERAMENT|G, BONESUPPORT AB, SWEDEN). After one year, a normal cortical thickness surrounding the defect was seen in approximately 80% of patients. Inside the defect-cavity, an almost complete product-resorption was seen after one year. The most common complication was a post-operative inflammatory soft-tissue reaction, seen in 7 patients (20%), which resolved without further treatment, although short-term antibiotic treatment was initiated at a local hospital in 6 patients, due to suspected wound infection. In summary, cortical thickness most commonly normalizes after bone tumor removal and filling of the bone defect using this particular composite ceramic bone graft substitute. The ceramic substitute undergoes resorption, which causes progressive changes in the radiological appearance inside the bone defect.

## Introduction

How to manage a bone-defect following bone-tumor surgery is controversial, and some authors believe that defect-grafting is only rarely required^1,2^. One of the main reasons for this controversy is the lack of objective clinical evidence regarding new-bone formation in bone-defects, and the effect on bone-strength. The evidence is obviously lacking due to heavy restrictions on what is possible to measure in humans. Perhaps the most important parameter, bone-strength, is virtually impossible to measure objectively. Cortical thickness is therefore typically used as a surrogate measure for bone-strength, and various criteria, incorporating cortical thickness, have been developed, in order to predict the risk of fracture^3,4^. Some authors have used the lack of post-operative fractures as an indirect measure for bone-strength^1,2^. Although this is intuitively reasonable, the question remains if cortical thickness in fact normalizes. A partially decreased cortical thickness might not cause problems for young and healthy individuals, but it could likely pose a problem for older and osteoporotic individuals. We therefore believe that efforts should be made to restore cortical, and to some extent cancellous, bone-volume, in order to regain and retain bone-strength. In this observational study, we aim to quantify changes in cortical thickness, as well as changes in the radiological appearance inside the defect cavity, following bone-defect filling, using a composite ceramic bone graft substitute (BGS), in the treatment of patients with bone-tumors and cysts. Further, we wish to report the clinical results and complications of this particular BGS. We hypothesize that cortical thickness and cavity-healing increases during the first postoperative year after curettage and filling with a commercially available BGS in patients suffering from benign or borderline bone lesions.

## Results

We identified 34 patients (M/F = 17/17, mean age = 32 (5–69) years), who were treated for 35 benign (n = 32) or borderline bone-lesions (giant cell tumors of bone (n = 2) and chondrosarcoma grade 1 (n = 1)) (Table 1). Approximately 3 quarters of the bone-lesions were situated in the lower extremity (27 of 35). The most common histo-pathological diagnosis encountered were simple bone-cysts (n = 9), followed by enchondromas (n = 8).

**Table 1:** Patient and bone lesion characteristics.

No. of patients/lesions	34/35
Female/Male	17/17
Age (range)	32 (5–69) years
Defect size (range)	15 (1.1–145) mL
Campanacci grade	
1	22
2	11
3	2
Histology (n = 35)	
Simple cysts*	9
Enchondroma	8
Bone infarction	3
Intraosseous ganglion	3
Aneurysmal bone cyst	3
Giant cell tumor of bone	2
Non-ossifying fibroma	1
Giant cell granuloma	1
Chondrosarcoma grade I	1
Non-specific reactive lesion	4

*Children/Adults: 5/4.

### Radiological evaluation of bone healing

**Figure 1:**
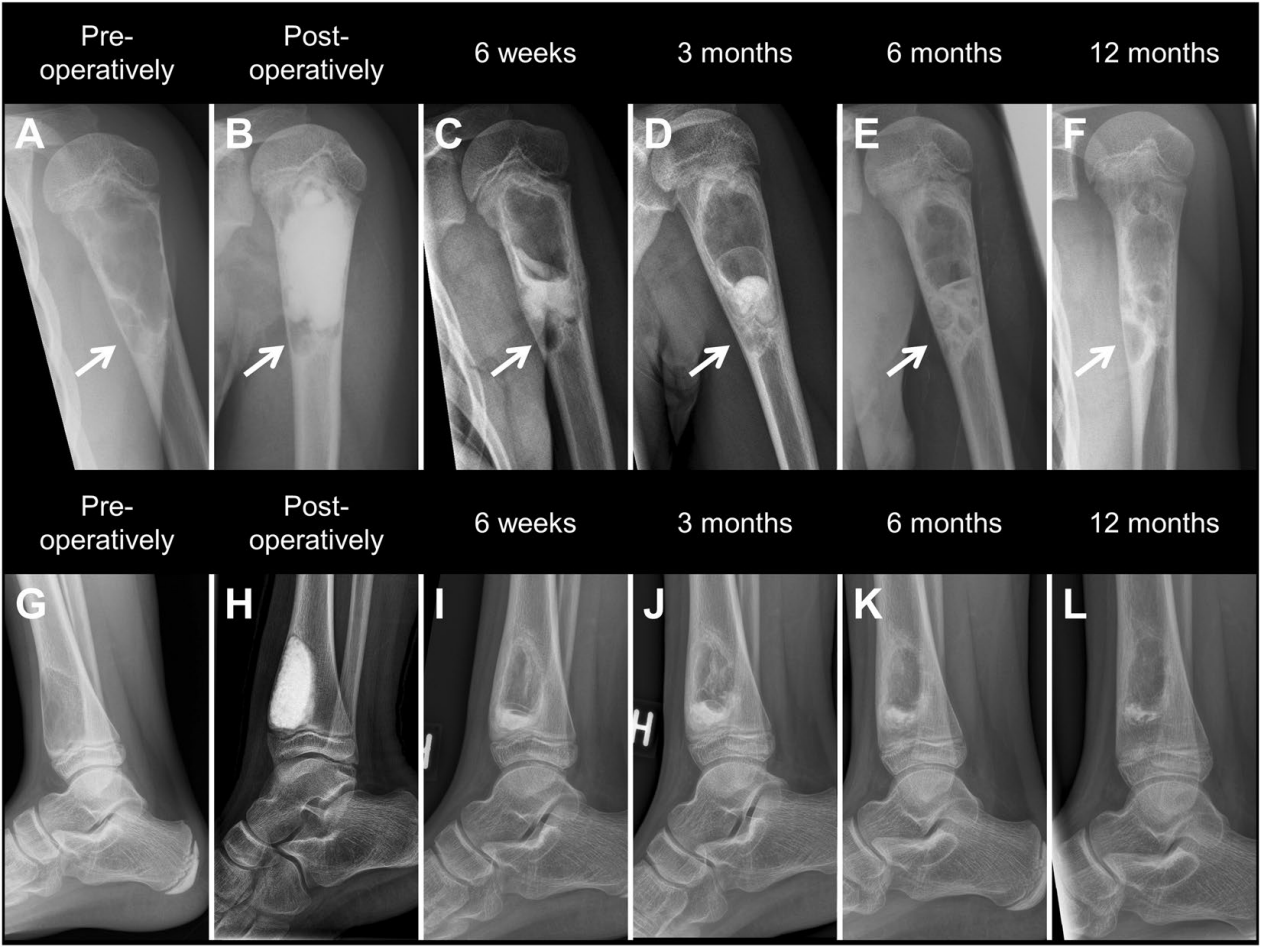
Upper row: Simple cyst of the proximal humerus treated by curettage and defect-filling using CERAMENT|BONE VOID FILLER. (**A**,**B**) Pre- and post-operative x-rays showing decreased cortical thickness as well an isolated chamber in the distal part of the affected bone (arrow), which is not accessed or filled during the operation. (**C–E**) X-rays taken after 6 weeks, 3 and 6 months showing increasing product resorption and cortical thickening. (**F**) X-ray taken after 12 months showing normal cortical thickness in the main part of the cyst, and reduced cortical thickness in the non-treated distal chamber as a sign of possible local recurrence. Lower row: Aneurysmal bone cyst of the distal tibia treated by curettage and defect-filling using CERAMENT|BONE VOID FILLER. (**G**,**H**) Pre- and post-operative x-rays showing decreased anterior cortical thickness and complete defect filling. (**I–L**) X-rays taken after 6 weeks and 3, 6, and 12 months showing increasing product resorption and cortical thickening. *Note the increasing distance between the growth-plate and the distal aspect of the cyst*.

**Figure 2:**
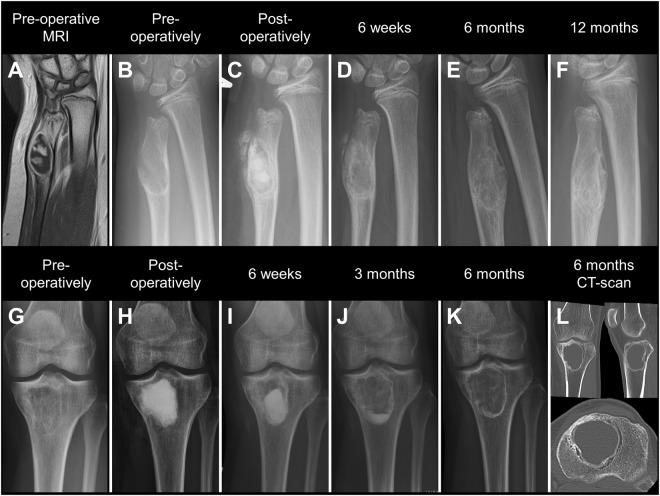
Upper row: Enchondroma of a 12-year old girl treated by curettage and defect-filling using CERAMENT|BONE VOID FILLER. (**A**,**B**) Pre-operative MRI and x-ray showing decreased cortical thickness. (**C**) X-ray taken post-operatively showing near complete defect-filling. (**D**,**E**) X-rays taken after 6 weeks, and 6 months showing increasing product resorption and cortical thickening. (**F**) X-ray taken after 12 months showing normal cortical thickness and signs of cavity-remodeling. Lower row: Large bone infarction of the proximal tibia treated by curettage and defect-filling using CERAMENT|BONE VOID FILLER. (**G**,**H**) Pre- and post-operative x-rays showing decreased cavity radiolucency and near complete defect-filling. (**I**–**K**) X-rays taken after 6 weeks, and 3 and 6 months showing increasing product resorption and persistent reduced defect-radiolucency. (**L**) 6-months CT-scan showing a peripheral reactive zone in the defect and a seemingly empty cavity.

Of the 35 bone lesions, 28 met the criteria for the comparative radiological evaluation at 12-months. The remaining 7 patients were excluded, due to local recurrence (n = 3) or re-operation before the 12-months control (n = 2), or due to missing 12-months x-ray (n = 2). Approximately 40% (11 of 28) of the defect-cavities showed normal radiological appearance after one year, whereas a normal cortical thickness was seen in 79% (22 of 28) of the cases (Table 2). In 13 patients, with a pre-operative reduction in cortical thickness, an improved thickness was achieved in 10 cases (77%) (Fig. 1). Only one patient, with a progressive lesion, had a diminished cortical thickness after one year. In 5 patients, all below 18 years of age, the ratio of normal-appearing bone inside the defect-cavity after one year improved in comparison to the ratio of post-operative defect-filling (Fig. 2, upper row). Conversely, the ratio of normal-appearing bone inside the defect-cavity was lower than the ratio of post-operative defect-filling in 12 patients.

**Table 2:** Comparative radiological evaluation.

Radiological appearance	Post-operatively	After 1 year	Grade development	
Cavity fill >90%	13^a^	11^b^	Improved	5
Cavity fill 50–90%	12^a^	8^b^	Same	11
Cavity fill 10–50%	3^a^	5^b^	Reduced	12
Cavity fill <10%	0^a^	4^b^		
Cortex thickness >90%^c^	15	22	Improved	10
Cortex thickness 50–90%^c^	6	3	Same	17
Cortex thickness 10–50%^c^	6	2	Reduced	1
Cortex thickness <10%^c^	1	1		

^a^Cavity filling of bone graft substitute.

^b^Cavity filling of bone with normal radiographic appearance.

^c^Cortical thickness compared to surrounding normal cortex.

Of the 35 bone lesions, 31 met the criteria for the modified Neer’s classification, which includes local recurrences (n = 3) (Table 3). Evaluation of the 1-year radiographs according to the modified Neer’s classification deemed: 11 patients in group I (Complete healing); 9 in patients in group II (Partial healing), 8 patients in group III (Persistent lesion), and 3 in group IV (Recurrent lesion). It should be noted that 6 of the 8 patients in group III were deemed to this category, due to incomplete defect fill, despite a normal, or close to normal, cortical thickness, while only 2 patients were in this category, due to significantly reduced cortical thickness.

**Table 3:** Modified Neer classification of radiological evaluation of bone defect healing.

Score	Classification	Description
I	Complete Healing	Complete or almost complete* filling of the initial lesion with radiological evidence of new bone formation
II	Partial Healing	Incomplete healing and/or graft resorption in an area(s) less than 50% of the initial lesion with enough cortical thickness to prevent fracture
III	Persistent Lesion	Graft resorption or persistent radiolucent area (s) greater than 50% of the initial lesion and/or with a thin cortical rim potentially at risk for fracture
IV	Recurrent lesion	Progressive lesion reappeared in a previously obliterated area or a residual radiolucent area verified by biopsy

*with or without small non-progressive radiolucent area(s) less than 1 cm in size.

### Local recurrence and reoperations

Four patients had local recurrence: 2 giant cell tumors, after 3 and 7 months (pathology verified); and 2 aneurysmal bone-cysts, after 3 months and 16 months (progression on x-ray) (Table 4**)**. The giant cell tumors were treated by re-curettage, while the aneurysmal bone-cysts were treated percutaneously by injection of a sclerosing agent (sclerotherapy). Two patients received re-curettage due to suspected recurrence of a lesion with a non-specific primary diagnose, but neither recurrence nor a more precise diagnose was confirmed. Three patients received a second grafting procedure in order to further increase bone stock, although no progression or recurrence was seen: two children with large bone cysts in the pelvis and proximal femur received re-injection of CERAMENT|BONE VOID FILLER: one filled percutaneously (pelvis) after 13 months, and one filled at the same time as a scheduled plate removal after 20 months; and one adult was treated with curettage and bone grafting after 10 months, due to a large remaining cavity just beneath the knee-joint (Fig. 2, lower row). One patient with an intra-osseous ganglion received re-curettage and bone-grafting after 14 months, due to persistent pain from the ankle joint, which was expected to resolve by complete cavity-filling, despite a normal cortical thickness.

**Table 4:** Re-operations.

#	Histology	Bone	Indication	Time-point*	Re-operation
1	GCT^a^	Distal tibia	Local recurrence	3 m	Curettage
2	GCT^a^	Proximal femur	Local recurrence	7 m	Curettage
3	ABC^b^	Proximal fibula	Local recurrence	3 m	Sclerotherapy
4	ABC^b^	Distal tibia	Local recurrence	16 m	Sclerotherapy
5	Non-specific	Proximal femur	Suspected LR^e^	21 m	Curettage
6	Non-specific	Patella	Suspected LR^e^	11 m	Curettage
7	SC^c^	Proximal femur	Plate removal	20 m	Inj CERAMENT
8	SC^c^	Pelvis	Pain/Bone stock	13 m	Inj CERAMENT
9	Bone infarction	Proximal tibia	Bone stock	10 m	Curettage
10	IOG^d^	Distal tibia	Pain	14 m	Curettage

^*^Time from primary operation (months).

^a^GCT: Giant cell tumor.

^b^ABC: Aneurysmal bone cyst.

^c^SC: Simple cyst.

^d^IOG: Intraosseous ganglion.

^e^LR: local recurrence.

In case of a suspected local recurrence (n = 6), the indication for reoperation was decided on a multidisciplinary team conference. Choice of defect-filling in the following re-operation, as well as indication for revision surgery in the remaining 4 cases, was based on clinical judgement of the attending physician.

### Postoperative complications and radiological irregularities

Minor complications occurred in 9 patients. One patient had a suspected post-operative non-displaced fracture of the medial malleolus, which was noticed retrospectively during a radiographic conference 2 months later. No intervention was needed. One patient had postoperative wound secretion for 10 days, which resolved by its own without further treatment or complications. Seven patients developed postoperative soft-tissue inflammation around the surgical site, which resolved without additional signs of wound-infection: such as wound secretion or pus. Despite this, 6 patients were prescribed prophylactic antibiotics, typically by their local emergency department or general practitioner. Of the 7 patients with an inflammatory reaction, 6 patients had a lesion in close relation to the knee, and one had a lesion in the foot. One of the patients, with an inflammatory reaction, was suspected of postoperative compartment-syndrome in the anterior and lateral muscle compartments of the lower leg, after defect-filling of the proximal fibula. Symptoms were pain, swelling, and impaired dorsi-flexion in the ankle joint and the patient received a fasciotomy, revealing intact muscle-tissue. After a sclerotherapy-injection 3 months later, in the same bone defect, the patient developed a transient post-injection peroneal palsy. The apparent proximity between the tumor and the peroneal nerve was suspected to have caused the impaired dorsi-flexion in relation to the primary operation as well, making the compartment-syndrome diagnosis less likely. No patients experienced a deep infection.

**Figure 3:**
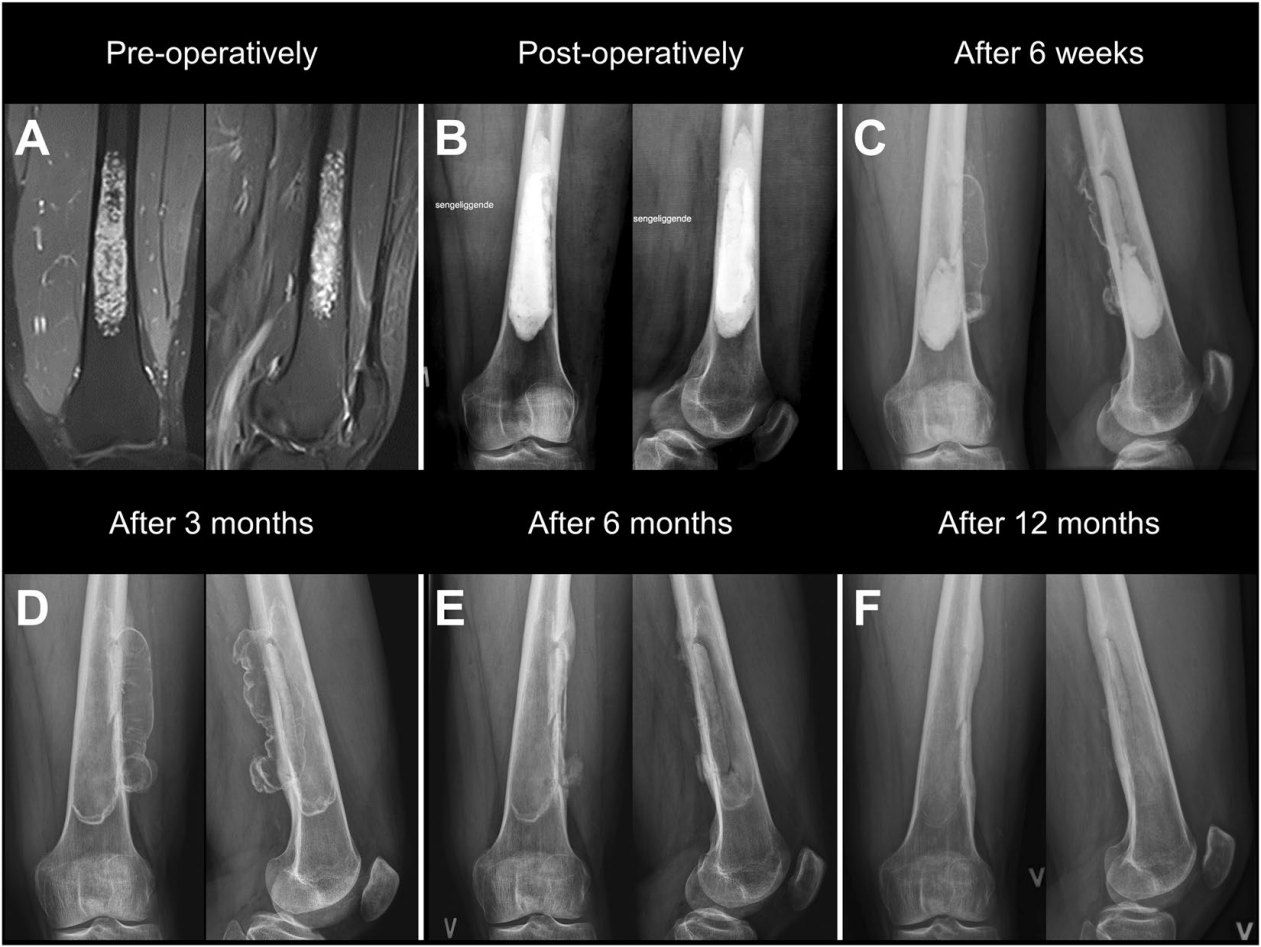
A patient treated by curettage and defect filling, using a combination of CERAMENT|G (20mL) and CERAMENT|BONE VOID FILLER (36mL) via an extended cortical window, for a large enchondroma in the left distal femur. (**A**) Pre-operative MRI-scan. (**B**) Post-operative x-ray without product-leakage (**C**,**D**) X-rays after 6 weeks and 12 weeks showing soft-tissue leakage. (**E**) X-ray after 6 months showing almost complete resorption of soft-tissue leakage. Outline of the bone window is clearly visible. (**F**) X-ray after 12 months showing complete resorption of the soft-tissue leakage, blurring of the bone window, and thickening of the posterior and lateral cortex.

In 9 patients, an irregularity was noticed on the follow-up x-rays. In 7 of 9 patients, a small amount of bone-graft substitute was seen in the soft-tissue on the x-ray taken immediately postoperatively. In all 7 cases, the bone-graft substitute resorbed within 3 to 6 months. Only 1 of the 7 cases developed an inflammatory reaction. One of 9 patients, with a bone-defect in the femur requiring a large bone window, developed a progressive leak of the bone-graft substitute into the soft-tissue, despite no leakage was seen on the postoperative x-rays (Fig. 3). The leak was non-symptomatic and resolved between 6 and 12 months postoperatively. One of 9 patients developed a calcification in the trochanteric bursa after treatment of a bone defect in the proximal femur. No leak of bone-graft substitute was seen in this patient.

## Discussion

In this study, we have quantified changes in cortical thickness and radiological bone cavity appearance, following tumor removal and bone defect filling using a composite ceramic bone graft substitute, in 35 benign bone-tumors or cysts. We found that cortical bone responded well to treatment. After one year, cortical bone thickness normalized, or remained normal, in 80% of cases. Inside the bone cavity, normal radiographic appearance was achieved in 40% of cases after one year. Only in patients, below the age of 18, did the degree of normal radiographic appearance, inside bone cavity, improve beyond the degree of postoperative defect-filling.

Prediction of risk factors for a pathological fracture has been studied mostly in patients with metastatic bone disease^5,6^. Although different criteria have been used, all studies agree that cortical impairment increases the risk of fracture. Direct translation of these results to benign bone lesions should be done carefully, but in a study of 155 benign bone cysts, similar results were shown^7^. The positive effect on cortical thickness, seen in our study, would therefore most likely have improved bone strength.

In terms of translating the radiological cavity-appearance into trabecular bone healing, the results should be interpreted cautiously. In some parts of the skeleton, up to 50% of the bone mass needs to be destroyed by an osteolytic lesion, before it is detectable on plain x-ray^8^. The ratio between cortical and trabecular bone varies greatly, depending on the bone-region, from around 50:50 in metaphyses of long bones down to 95:5 in diaphyseal bone^9^. This means that evaluation of trabecular bone formation is virtually impossible on plain radiographs, as most changes will be invisible due to overlapping cortical bone, especially in diaphyseal bone.

Kaczmarczyk *et al*. evaluated the same bone graft substitute, as used in our study, for percutaneous treatment of bone defects^10^. They found complete healing (grade 1), according to a modified Neer’s classification, in 11 of 14 patients, despite incomplete post-operative filling in most cases. Possible explanations for the higher rating compared to our study include: difference in mean age (13 years compared to 32 years in our study), composition of histology (8 of 14 were simple cysts in the proximal humerus), and tumor localization. Low age was associated with an improved radiological cavity-appearance in our study, and the typical localization of proximal humeral bone cysts, in the transition between metaphyseal and diaphyseal bone, makes trabecular bone loss difficult to assess, due to a thick cortical background.

Findings of characteristic radiological changes inside the bone cavity, due to material resorption, are shared by many studies evaluating this product^10–12^. This is likely due to resorption of the calcium-sulphate component, because pure calcium-sulphate-based products are known to undergo rapid resorption^13,14^. Although partial resorption of CERAMENT is intended, to allow room for bone ingrowth, the rate of resorption could, unintendedly, have contributed to a higher number of re-operations in our study. We observed 10 re-operations: 5 within the first year and 5 in the second year. Product resorption did *not* influence the indication for re-operation in the 4 patients having a local recurrence, in tumor-entities with a knowingly high local recurrence rate^15,16^; nor in the 2 patients, where CERAMENT was re-injected. However, in the remaining 4 patients, the pattern of resorption of this product could have been a contributing factor in the decision to perform a re-operation. Firstly, it can be difficult to distinguish between the natural pattern of resorption and resorption due to disease progression. This uncertainty might have contributed to re-operation of two patients were primary operation had failed to give a specific diagnose. Secondly, the rate of product-resorption extinguished the hope of restoring normal trabecular bone throughout the cavity in two patients. And this was a central factor in the decision to make a re-operation, although cortical thickness was sufficient to permit full weight bearing (Fig. 2).

Beside a fast rate of resorption, calcium sulphate-containing bone graft substitutes are known to cause prolonged wound secretion^13,17^. Our typical wound closure technique, including intradermal suture and a skin glue, might explain why we only observed prolonged wound secretion in one patient. The inflammatory reaction seen in our cohort has previously been described in a study evaluating the same bone-graft substitute for defect-filling in hand-surgery^12^. The anatomical region might be a factor to determine which patients develop this inflammation, because it has only been described in areas with little soft-tissue coverage, i.e. the knee, foot and hand. Although the inflammation seems benign and only causes temporary symptoms, it can lead to excessive use of antibiotics, when symptoms are presented to doctors not familiar with this phenomenon. In the previously mentioned study by Kaczmarczyk *et al*., no such complications were encountered^10^. The percutaneous technique used in their study could explain the low complication rate, possibly due to a low amount of product leakage into the soft-tissue. Considering that none of the patients in our study developed a deep infection, the inflammatory reaction is most likely aseptic. However, the absence of infections could also have been influenced by the local antibiotic delivery by the BGS.

The main limitations of our study are, firstly, the lack of 3-dimensional quantifications of bone formation. But due to ethical restrictions, repeated CT scans could not be performed in this study. However, cortical thickness is one of the parameters, which can be reliably evaluated in conventional x-rays, due to the high mineral density in cortical bone. Conversely, we could not reliably quantify the formation of new trabecular bone, but only describe the radiological appearance of the defect cavity. Secondly, this study is observational without any control group, which makes it impossible to distinguish between the effect of natural bone formation and the effect of the bone-graft substitute. However, we do get an indication of an actual effect of the bone-graft substitute in one case, where a patient with a bi-cameral bone-cyst acted as her own control group. One of the chambers, not receiving any filling, did not show any signs of healing, while the other chamber showed a good response to treatment (Fig. 1, upper row).

In conclusion, cortical thickness most commonly normalizes after bone tumor removal and filling of the bone defect using this particular composite ceramic bone graft substitute. The ceramic material undergoes resorption, which causes progressive changes in the radiological appearance inside the bone defect. This radiological behavior should be kept in mind to ensure alignment of pre-operative expectations and the surgical result. Soft-tissue inflammation is a complication to the use of this bone graft substitute, but the reaction seems to be self-limiting. Otherwise, the use of this product has a low complication-rate.

## Materials and Methods

### Study Design

We performed a prospective study of all consecutive patients, who received bone-defect filling using two variants of the same composite ceramic bone graft substitute (CERAMENT|BONE VOID FILLER or CERAMENT|G, BONESUPPORT, SWEDEN) in the treatment of benign or borderline bone-tumors and cysts, in our orthopedic oncology department between August 1^st^ 2014 and March 1^st^ 2016. During this period, the bone graft substitute was first choice for bone-defect filling. All relevant data (age, gender, histological diagnosis, grade, size and anatomical location of the lesion, details of the surgical procedure, use of orthopaedic implants, and occurrence of postoperative complications or local recurrence) were collected from the medical records, the national pathology database^18^, and the country-wide electronic patient file system. At the latest follow-up (May 1^st^ 2017) the minimum follow-up was 14 months (mean 22 (14–30) months).

### Bone-Lesion Characteristics and Surgical Intervention

We calculated lesion-size by approximating measures from CT/MRI to a spherical shape (Height × Width × Length × 0.52)^1^. The cortical bone around the lesions was graded, with respect to radiological appearance, according to a *modified Campanacci-classification*, as intact (Grade 1), thinned (Grade 2), or breached by the tumor (Grade 3)^19^.

The indication for surgery was most often to obtain diagnosis (n = 23), and the remaining indications were: impending fracture (n = 4), progressive benign cyst (n = 4), and local recurrence (n = 4). Open curettage was performed in 31 cases, while an entirely percutaneous procedure was performed in 4 cases. CERAMENT|BONE VOID FILLER was used in the majority of cases (27 of 35), CERAMENT|G in 6 of 35 cases, and a combination of both in 2 of 35 cases. The mean amount of BGS used was 13 (2–56) mL. In 3 larger lesions, deemed structurally impaired or at-risk of fracture, mechanical augmentation using plates was performed in addition to the bone-defect filling.

### The composite ceramic bone-graft substitute

The two variants of the BGS have the same mineral composition and consist of 60% calcium-sulphate and 40% hydroxyapatite. Calcium-sulphate has a fast rate of resorption, typically within the first 6–12 weeks after the operation^20,21^. CERAMENT|BONE VOID FILLER contains the contrast-agent, Iohexol, for enhanced radio-opacity. CERAMENT|G does not contain Iohexol, but contains 175 mg of gentamicin per 10 mL paste^22,23^. Despite the mentioned differences, the two products are expected to have similar mechanical and degradation properties due to their similar mineral composition^22,23^.

### Radiological evaluation

X-rays of the bone defects were evaluated by a specialist in radiology and two senior orthopedic surgeons. The post-operative x-rays were assessed for cortical thickness and the ratio of defect filling by the BGS. The x-rays taken one year after the operation were assessed for cortical thickness and the ratio of normal-appearing bone within the cavity (same opacity as surrounding bone). The pre-operative and 1-year x-rays where compared for cortical thickness and cavity filling (Table 2**)**. In the table showing the comparative radiological evaluation, the ratio of normal-appearing bone is referred to as *cavity filling*. In order to simplify the process of quantification, both cavity filling and cortical thickness were categorized into 4 groups: (1) >90% fill/thickness; (2) 50–90% fill/thickness; (3) 10–50% fill/thickness; and (4) <10% fill/thickness. The cortical-thickness-ratio was calculated by comparison to unaffected surrounding cortical bone.

In addition to the quantitative evaluation, x-rays taken after 1 year were classified according to a modified Neer’s classification of radiological bone-cyst appearance (Table 3)^10,24^. Patients, with/or suspected of local recurrence *within* 12 months of the primary operation, were classified as *local recurrence* in the modified Neer’s classification, although they did not achieve a full 12-months radiological follow-up, due to reoperations.

### Study approval and informed consent

Study approval was obtained from the Danish Data Protection Agency (J.no. 2014-41-3021) and the Department of Health Agency. The study was performed in accordance with relevant guidelines and regulations. Written informed consent to participate in the study was obtained from the patients or their parents.

## Data Availability

The authors do not have permission to share data from this study.
